# Malaria Parasites Hijack Host Receptors From Exosomes to Capture Lipoproteins

**DOI:** 10.3389/fcell.2021.749153

**Published:** 2021-11-11

**Authors:** Naoyuki Iso-o, Keisuke Komatsuya, Fuyuki Tokumasu, Noriko Isoo, Tomohiro Ishigaki, Hiroshi Yasui, Hiroshi Yotsuyanagi, Masumi Hara, Kiyoshi Kita

**Affiliations:** ^1^ The Institute of Medical Science, The University of Tokyo, Tokyo, Japan; ^2^ Department of 4th Internal Medicine, Teikyo University Mizonokuchi Hospital, Kawasaki, Japan; ^3^ Department of Biomedical Chemistry, Graduate School of Medicine, The University of Tokyo, Tokyo, Japan; ^4^ Laboratory of Biomembrane, Tokyo Metropolitan Institute of Medical Science, Tokyo, Japan; ^5^ Department of Lipidomics, The University of Tokyo, Tokyo, Japan; ^6^ Department of Cellular Architecture Studies, Institute of Tropical Medicine, Nagasaki University, Nagasaki, Japan; ^7^ Department of Physiology, Teikyo University School of Medicine, Tokyo, Japan; ^8^ School of Tropical Medicine and Global Health, Nagasaki University, Nagasaki, Japan; ^9^ Department of Host-Defense Biochemistry, Institute of Tropical Medicine, Nagasaki University, Nagasaki, Japan

**Keywords:** malaria, plasmodium falciparum, cholesterol, lipoproteins, lipid, exosomes, scavenger receptors

## Abstract

Malaria parasites cannot multiply in host erythrocytes without cholesterol because they lack complete sterol biosynthesis systems. This suggests parasitized red blood cells (pRBCs) need to capture host sterols, but its mechanism remains unknown. Here we identified a novel high-density lipoprotein (HDL)-delivery pathway operating in blood-stage *Plasmodium*. In parasitized mouse plasma, exosomes positive for scavenger receptor CD36 and platelet-specific CD41 increased. These CDs were detected in pRBCs and internal parasites. A low molecular antagonist for scavenger receptors, BLT-1, blocked HDL uptake to pRBCs and suppressed *Plasmodium* growth *in vitro*. Furthermore, platelet-derived exosomes were internalized in pRBCs. Thus, we presume CD36 is delivered to malaria parasites from platelets by exosomes, which enables parasites to steal HDL for cholesterol supply. Cholesterol needs to cross three membranes (RBC, parasitophorous vacuole and parasite’s plasma membranes) to reach parasite, but our findings can explain the first step of sterol uptake by intracellular parasites.

## Introduction

Cholesterol is an important lipid for cellular homeostasis and cell membranes and it is required for protein functioning and lipid asymmetry (Rog and Vattulainen, 2014; Steck and Lange, 2018). Therefore, it is intriguing that no evidence exists for complete *de novo* sterol biosynthesis in *Plasmodium* (the malaria-causing apicomplexan parasite (Ormerod and Venkatesan, 1982; Vial et al., 1984; Wunderlich et al., 1991; Haldar et al., 2002; Rohrich et al., 2005; Ehrenman et al., 2013)), despite pRBCs containing less cholesterol than normal erythrocytes (Lambrecht et al., 1978; Maguire and Sherman, 1990; Wunderlich et al., 1991). Cholesterol is an essential nutrient for parasite growth and characteristic cholesterol concentration gradient is established in parasitized erythrocytes (Tokumasu et al., 2014). Cholesterol in erythrocyte membrane is sorted into the internal malaria parasite (Hayakawa et al., 2020) and a few reports have indicated that Niemann-Pick C1-like 1 (NPC1L1) is involved in cholesterol uptake in apicomplexans (Ehrenman et al., 2013; Istvan et al., 2019). These reports suggest that some protozoans acquire host sterols, raising the prospect that sterol supply to them may be a potential therapeutic target in these pathogens. An association between cholesterol and malaria parasites, as reported in a patient-based study, showed that high density lipoprotein (HDL) levels in serum decreased markedly in patients with *Plasmodium vivax* infections (Lambrecht et al., 1978). While malaria parasites could obtain sterols in various ways, blood-stage *Plasmodium* species possibly take up lipoproteins as a sterol source. However, the known major lipoprotein receptors, including class B scavenger receptors (SR-Bs), are generally scarce on mature erythrocyte surfaces (Edelman et al., 1986; Kieffer et al., 1989; Greenwalt et al., 1992). Additionally, no candidate genes have been identified in the *P. falciparum* (*Pf*) genome that appear to be mammalian SR-Bs or low density lipoprotein (LDL) receptor family orthologues (Watanabe et al., 2001). These findings indicate that foreign lipoprotein receptors are required for lipoprotein delivery to pRBCs during parasite growth. In this study, we explored possibilities for the lipoprotein receptors on the pRBCs and found that host exosomes delivered CD36 selectively to the pRBC membranes.

## Materials and Methods

### *P. berghei* and Host Animals, Survival Times and Parasitemia

To investigate the time-series behavior of CD36, 1 × 10^7^ ANKA strain *P. berghei* parasitized mouse erythrocytes were intraperitoneally injected into male CD36^−/−^ mice and congenic C57BL6 mice (The Jackson Laboratory, Bar Harbor, ME, United States), both at 12 weeks of age. Health was monitored every 12 h, and blood sampling was performed from tail veins every 48 h. Parasitemia was estimated by Giemsa-stained thin blood smears. Statistical analysis of survival was performed using the log-rank test, and parasitemia was assessed using the Student’s *t*-test.

### *In vitro P. falciparum* Cultures

*P. falciparum* (3D7 line) was cultured at 3% hematocrit with type A human erythrocytes in RPMI 1640 medium (Thermo Fisher Scientific, Waltham, MA United States), supplemented with 25 mM NaHCO_3_, 10 μg/ml hypoxanthine, 25 mM HEPES, 0.8 mg/mL l-glutamine, 40 μg/ml gentamicin sulphate, and inactivated 5% type A human serum (Trager and Jensen, 1976). Cultures were maintained under 5% O_2_, 5% CO_2_, and 90% N_2_ at 37°C. Parasitemia was estimated from thin blood smears stained with Giemsa. The experiments using human erythrocytes were performed under the guidelines of the ethics committee of The University of Tokyo (#10050 and #11064). Human erythrocytes were obtained from the Japan Red Cross Society (No: 28J0058). Parasites were synchronized using 5% D-sorbitol treatment for 10 min to obtain ring stages.

### Fluorescence Microscopy

Erythrocytes were crosslinked using 50 mM dimethyl suberimidate (DMS) (MilliporeSigma, Burlington, MA, United States) in 100 mM sodium borate buffer, pH9.5, containing 1 mM MgCl_2_ for 1 h (Tokumasu and Dvorak, 2003). An additional fixation was performed using 2% paraformaldehyde in phosphate-buffered saline (PBS) for 10 min. The reaction was quenched using 0.1 M glycine in PBS for 1 h. Erythrocytes were blocked in 3% bovine serum albumin (BSA), 0.2% Tween 20 in PBS for 1 h and incubated with an anti-CD36 antibody (diluted 1:200) (Proteintech Group Inc., Rosemont, IL) in 1% BSA, 0.2% Tween 20 in PBS. Erythrocytes were washed in 0.2% Tween 20 in PBS three times and then incubated with Alexa Flour 488-conjugated goat anti-rabbit IgG (1:1,000) for 1 h and Hoechst 33,258. The images were captured by Zeiss LSM780, a confocal microscopy system.

### Fluorescent Assay for HDL Uptake *via* CD36

SR-B1-deficient HuH-7 cells expressing CD36 were provided by Osaka University (Yamamoto et al., 2016). They were seeded confluently on 96-well plates (Fluoro Nunc Black Plate, Thermo Fisher Scientific) and then preincubated for 1 h with different concentrations of BLT-1 (MilliporeSigma) in DMEM supplemented with 10% lipoprotein-deficient human plasma, that was obtained after LDL and HDL were isolated using a conventional ultracentrifugation method (Havel et al., 1955), and dialyzed against PBS. The cells were incubated for 8 h with 100 μg/ml DiO-labelled HDL. After washing in DMEM containing 0.2% BSA three times, the DiO signal was measured using FLUOstar OPTIMA microplate reader (BMG LABTECH, Ortenberg, Germany) at 485 or 515 nm for excitation or emission, respectively.

### Lipoprotein Purification and Labelling

Human LDL and HDL were isolated from human plasma using a conventional ultracentrifugation method (Havel et al., 1955) followed by dialysis against PBS. Fluorescence lipid indicator DiI (1,1′-dioctadecyl-3,3,3′,3′-tetramethylindocarbocyanine perchlorate, Thermo Fischer Scientific) and DiO (3,3′-dioctadecyloxacarbocyanine perchlorate, Thermo Fischer Scientific) labelling was conducted as described previously (Pitas et al., 1992; Fisher et al., 2007). CE-BODIPY (CholEsteryl 4,4-Difluoro-5-(4-Methoxyphenyl)-4-Bora-3a,4a-Diaza-s-Indacene-3-Undecanoate, Thermo Fisher Scientific) labelling was performed following the method of Reaven with modifications (Reaven et al., 1995). Briefly, the HDL3 fraction (specific gravity (SG) 1.125–1.21) was isolated from total HDL, and 6.3 mg of HDL_3_ protein was mixed with 0.1 μmol of CE-BODIPY dissolved in 0.2 ml of RPMI 1640. The preparation was sonicated using BIORUPTOR 402 UCD-200TM (Cosmo Bio Co., Ltd., Tokyo Japan) at 200 W for 20 min at room temperature (RT). The reconstituted HDL was isolated using sequential ultracentrifugation with SG between 1.065 and 1.21, followed by dialysis against PBS.

### Lipoprotein Uptake by *P. berghei* and *P. falciparum*

Erythrocytes collected from *Pb*-parasitized BALB/c male mice at 80% parasitemia were incubated under 20% O_2_, 5% CO_2_, and 75% N_2_ gas at 37 °C for 1.5 h in RPMI 1640 media with 100 μg/ml of LDL or HDL. Erythrocytes were washed with RPMI 1640 media containing 0.2% BSA, and then analyzed by fluorescence activated cell sorting (FACS). To study the effect of scavenger receptor blockade using BLT-1, erythrocytes were preincubated in complete RPMI 1640 media with BLT-1 for 0.5 h, followed by addition of labelled HDL, and re-incubation for 1.5 h. Erythrocytes were washed with RPMI 1640 media containing 0.2% BSA, and then analyzed by fluorescence activated cell sorting (FACS) (FACS Calibur, Becton, Dickinson and Company, Franklin Lakes, NJ, United States) or the wet mounts were observed by confocal microscopy (Type A1Rsi, Nikon, Nikon Instec. Co., LTD., Tokyo Japan). *Pf* parasite cultures included 100 μg/ml DiI-labelled lipoprotein (24 h, 37°C). Erythrocytes were washed with RPMI 1640 media containing 0.2% BSA, and then observed by fluorescent microscopy (Axio ImagerM2, ZEISS, Oberkochen, Germany).

### Erythrocyte Membrane Fraction Preparation

To minimize platelet contamination, mouse whole blood collected with EDTA was first centrifuged under a 1.063-density barrier of OptiPrep (AXIS-SHIELD) (350 × *g*, 15 min). The supernatant was removed and the pellet was washed with PBS and passed through a Plasmodipur Filter (Euro Proxima, Arnhem, Netherlands) for leucocyte removal. Purified erythrocytes were hemolyzed in 5 mM phosphate buffer (19 ml of 5 mM NaH_2_PO_4_ plus 81 ml of 5 mM Na_2_HPO_4_, pH 7.4), and then centrifuged (12,000 × *g*, 10 min). The pellet was solubilized in radioimmunoprecipitation assay (RIPA) buffer (25 mM Tris-HCl pH 7.6, 150 mM NaCl, 5 mM EDTA, 1% Triton X-100, 1% sodium deoxycholate, 0.1% SDS) as the membrane fraction, and the supernatant was retained as the cytosol fraction with storage at –20°C. These samples were mixed with sample buffer containing 10% 2-mercaptoethanol and boiled at 95°C before SDS electrophoresis.

### Mouse Platelet Preparation

Mouse whole blood was collected with a 10% volume of ACD-A (anticoagulant citrate dextrose solution-A) solution and centrifuged (200 × *g* for 12 min and 500 × *g* for 12 min at 4°C). Prostaglandin E1 (100 ng/ml; Santa Cruz Biotechnology, Inc., Dallas, TX, United States) was added to the supernatant followed by centrifugation (2,700 × *g*, 15 min). The pellet was solubilized in RIPA buffer and stored at −20°C.

### Exosome Isolation From Mouse Plasma

After platelet collection, the residual plasma was centrifuged (20,000 × *g*, 40 min) to eliminate debris and microvesicles. The supernatant was diluted 1:7 in PBS and ultracentrifuged (100,000 × *g*, 1 h, 4 °C). The pellet was suspended in PBS and recentrifuged (100,000 × *g*, 1 h). After suspension in PBS (2.5 μl of PBS for exosomes from 100 μl of plasma), the suspension was rapidly frozen in liquid N_2_ and stored at −80°C.

### Isolating the Exosomes Released From CMK11-5 Cells

CMK11-5 cells, suspended at 1.0 × 10^6^ cells/mL in RPMI 1640 media, were preincubated with 1.0 × 10^–7^ M phorbol-12-myristate-13-acetate (MilliporeSigma) for 30 min for cell differentiation, and then cultured using RPMI 1640 media supplemented with 10% exosome-depleted fetal bovine serum (System Biosciences, LLC., Palo Alto, CA, United States) for 96 h CaCl_2_ (final Ca^2+^ concentration, 2.0 mM) and 10 μM Ca^2+^ ionophore A23187 (MilliporeSigma) were added and incubated for further 30 min. The culture medium was harvested, passed through a 0.22-μm micropore filter and ultracentrifuged twice (100,000 × *g*, 1 h) to isolate the exosomes. To fluorescently label the exosomes, 20 ml of filtered culture media plus the exosomes was incubated with 5 μl of DiI (2.5 mg/ml in DMSO) (3 h, 37°C). The DiI-labelled exosomes were isolated by sequential ultracentrifugation steps as described above.

### *Ex vivo* Study on CD36 and PECAM-1 Delivery From CMK11-5-Derived Exosomes to the *Pb*-Infected Mouse Erythrocytes

The erythrocytes from 12-week-old CD36^−/−^ male mice (for CD36) or C57BL6 mice (for PECAM-1), 7 days after *Pb* infection (1.0 × 10^7^ cells) were incubated with non-labeled exosomes (0.15 mg of protein) that were suspended in 100 μl of RPMI1640 media, and kept at culture condition for 6 h. Erythrocytes were washed three times with RPMI 1640 media containing 0.2% BSA, and suspended in 5% BSA in PBS. Smears were prepared from them for further analyses.

### *Ex vivo* Study on CMK11-5-Derived Exosome Trafficking Into Parasitized Mouse Erythrocytes

DiI-labelled exosomes (0.15 mg protein) were suspended in 100 μl of RPMI 1640 media, and erythrocytes from 12-week-old C57BL6 male mice, 7 days after *Pb* infection (1.0 × 10^7^ cells) were incubated in this media, under 20% O_2_, 5% CO_2_ and 75% N_2_ mixed gases at 37°C for 1 h. Erythrocytes were washed three times with RPMI 1640 media containing 0.2% BSA and then observed by confocal microscopy (Type A1Rsi, Nikon) as wet mounts.

### Bone Marrow Transplantation

Donor male CD36^−/−^ mice (7-week-old) were sacrificed and bone marrow tissue was immediately aspirated from their femoral bones and suspended in PBS. The 7-week-old recipient male Kusabira-Orange mice were X-ray irradiated (total 9.4 Gy) twice a day, and 5 × 10^5^ bone marrow cells were injected via the tail veins. At 5 weeks post-transplantation, KuO fluorescence was confirmed to be absent from the peripheral blood cells, including the platelets.

### Immunoblot Analysis

Samples (20 μg of total protein unless otherwise noted) of mouse erythrocytes, platelets, liver tissue, or exosomes were applied to 12.5% SDS-polyacrylamide gels (SuperSep Ace, FUJIFILM Wako Pure Chemical Corp., Osaka, Japan), electrophoresed and then transferred to PVDF membranes (BioRad) using a Trans-Blot SD transfer cell (Bio-Rad Laboratories, Inc., Hercules, CA, United States). Membranes were blocked with 5% fat-free milk in PBS (RT, 0.5 h) and reacted first with primary antibodies (Abs) in 2.5% fat-free milk in PBS (4°C overnight), followed by incubation with biotin-conjugated secondary Ab in 2.5% fat-free milk in PBS (RT, 1 h). Next, peroxidase-conjugated avidin-biotin complex (ABC standard kit, Vector Laboratories, Inc., Burlingame, CA) was added (RT, 0.5 h). Signals were detected using ECL Prime Western Blotting Detection Reagent (GE Healthcare) as the substrate and Amersham Imager 600 (GE Healthcare, Chicago, IL, United States) as the detector.

#### Primary Abs

Rabbit anti-CD36 polyclonal IgG (Proteintech) (1:500 dilution), rabbit anti SR-B1 monoclonal IgG (ab52629, Abcam, Cambridge, United Kingdom) (1:2000 dilution), rabbit anti-CD41 polyclonal Ab (ab63983, Abcam) (1:500 dilution), rabbit monoclonal anti-ALIX (ab186429, Abcam) (1:1,000 dilution), rabbit anti-Apo A I polyclonal Ab (reactive to both mouse and human Apo AI, ab33470, Abcam) (concentration, 1.5 μg/ml) and rabbit anti-CD31 or PECAM-1 polyclonal IgG (Proteintech Group Inc., Rosemont, IL) (1:500 dilution) were used in this study.

#### Secondary Ab

Biotin-conjugated goat anti-rabbit IgG heavy and light chains (ab97073, Abcam) were reacted against the primary Abs (1:10,000 dilution).

### Immunocytochemical Analysis

Mouse whole blood smears were air-dried and fixed in methanol. Samples from Kusabira-Orange mice and controls were fixed in 3% paraformaldehyde and quenched in 10 mM glycine-PBS. Samples were blocked using 5% BSA/PBS at (RT, 0.5 h), then reacted with primary Abs (4°C overnight), followed by the secondary Ab (RT, 1 h). Nuclear staining using bisBenzimide H33258 (MilliporeSigma) was employed where necessary.

#### Primary Abs

The anti-CD36 Ab (rabbit polyclonal IgG, Proteintech) was used at a 1:50 dilution in 1% BSA/PBS for single staining, and the rat anti-CD36 monoclonal IgG (R and D Systems, Inc., Minneapolis, MN, United States) was used at 20 μg/ml for the paired one-time staining with AMA-1 or CD41. Anti-SR-B1 rabbit monoclonal IgG was also used at a 1:50 dilution. Rabbit anti-*P. yoelii* AMA-1 polyclonal antiserum (Mutungi et al., 2014) was used at a 1:100 dilution, rabbit anti-CD41 polyclonal Ab (ab63983, Abcam) was used at a 1:50 dilution, and rabbit anti-CD31 or PECAM-1 monoclonal IgG (reactive to both mouse and human PECAM-1, ab182981, Abcam) was used at a 1:500 dilution.

#### Secondary Abs

Anti-rabbit IgG heavy and light chains conjugated to Alexa Fluor 647 (ab150083, Abcam) were used at a 1:200 dilution in 1% BSA/PBS, anti-rabbit IgG heavy and light chains conjugated with FITC (ab6717, Abcam) were used at a 1:1,000 dilution, and anti-rat IgG heavy and light chains conjugated to Texas Red (ab6843, Abcam) were reacted at a 1:2,500 dilution against each primary Ab.

### Sandwich ELISA

Rat monoclonal anti-CD41 Ab (NBP1-43415, Novus Biologicals, Centennial CO, United States) (100 μl, 1 μg/ml dilution) in NaHCO_3_/Na_2_CO_3_ buffer (50mM, pH 9.6) was incubated overnight at 4°C and fixed on each well of a 96-well immunoplate as the capture Ab. Exosomes from 50 μl of equally pooled plasma from five mice per group were sequentially incubated at room temperature for 1 h with goat polyclonal anti-CD36 Ab (AF2519, R and D Systems) and HRP-labelled bovine anti-goat IgG heavy and light chains, known to be minimally cross-reactive with mouse and rat serum proteins (805–035-180, Jackson ImmunoResearch, West Grove, PA, United States). Anti-CD36 and anti-goat IgG Abs were diluted to 0.4 μg/ml and 0.3 μg/ml, respectively, in PBS with 0.5% BSA, and 100 μl of each was added to each well. Final detection was performed using 100 μl of TMB substrate (BD Biosciences, San Jose, CA, United States) and 50 μl of 2M H_2_SO_4_. OD450 nm was measured using FLUOstar OPTIMA microplate reader. Washing in each step was performed with PBS without any detergent. ELISAs were performed as duplicate assays.

### Flow Cytometric Analysis

DiO-labelled HDL uptake was investigated using FACS Calibur or Canto II (BD biosciences). Erythrocytes collected from *Pb*-parasitized BALB/c male mice at 80% parasitemia were incubated under 20% O_2_, 5% CO_2_ and 75% N_2_ gas at 37°C for 1.5 h in RPMI 1640 media with 100 μg/ml of LDL or HDL. Erythrocytes were washed with RPMI 1640 media containing 0.2% BSA, and then analyzed by fluorescence activated cell sorting (FACS). DiO fluorescence was detected on the FL-1 channel. Parasitized RBCs were identified by SYTO 63 staining (Thermo Fisher Scientific) and gated as necessary. List mode data were analyzed using CellQuest software for Calibur and FlowJo software for Canto II.

## Results

### Lipoprotein Uptake by Parasitized Erythrocytes

**FIGURE 1:**
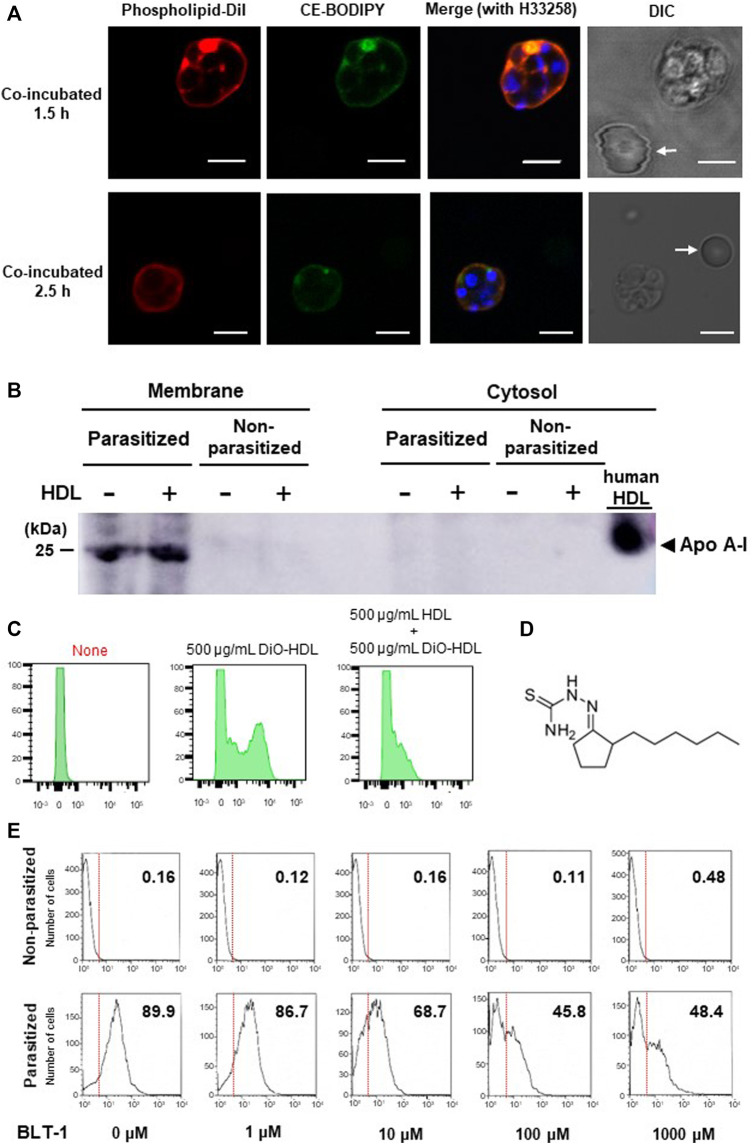
HDL-uptake by *Plasmodium*-infected erythrocytes. **(A)** Uptake of HDL components by *P. b*erghei-pRBCs. Phospholipid and ester components were concomitantly observed around parasite, but HDL was not taken up by the nRBCs (arrows). Scale bar: 5 μm.**(B)** *Pb*-pRBCs western blotted for Apo A-I, using a polyclonal primary Ab reactive to both mouse and human Apo A-I. The membrane fraction showed Apo A-I positivity with or without the addition of human HDL. **(C)** Flow cytometric analysis of DiO-labelled HDL uptake. Synchronized ring-stage *P. falciparum* incubated for 24 h with 500 μg/ml of HDL. HDL uptake was competitively inhibited by unlabeled HDL. **(D)** Chemical structure of BLT-1. **(E)** BLT-1 dose-dependent inhibition of DiO-labelled HDL uptake to *Pb*-pRBCs. The numbers in panels represent fraction (%) of DiO-positive cells out of total cells.

To test whether pRBCs can take up lipoproteins, we collected pRBCs from *P. berghei* (*Pb*)-infected BALB/c mice and incubated them with fluorescently-labelled lipoproteins. Fluorescence lipid indicator DiI targets phospholipid components, and CE-BODIPY targets cholesteryl ester components. HDLs with these indicators were both taken up by the pRBCs, but not by non-parasitized ones (nRBCs) (Figure 1A). Although the occasional local concentrations of the dye show the bright spots, the fluorescent signals from DiI and BODIPY showed similar patterns in the schizonts’ bodies, suggesting that these two HDL components were taken up together. Western blots showed apolipoprotein A-I (Apo A-I) signal-positivity from the membrane fractions of the pRBCs (Figure 1B), and the pRBCs were also Apo A-I-positive, with or without addition of human HDL, suggesting that it had been incorporated before blood sampling from the infected mice. HDL uptake in *Pf*-parasitized human erythrocytes was also apparent, and larger HDL amounts appeared to be taken up by schizont-pRBCs than by ring-parasitized ones (Supplementary Figure 1B), but its uptake was competitively inhibited by non-labelled HDL (Figure 1C). Contrastingly, flow cytometry analysis showed that LDL signals compared to non-parasitized erythrocyte remained the same, while HDL signals increased by infection, suggesting that LDL was not taken up by pRBCs (Supplementary Figure 1A). Known mammalian receptors that mediate HDL uptake but not native LDL uptake are SR-Bs such as CD36 or scavenger receptor class B type 1 (SR-B1) (Acton et al., 1994). The signal distribution of fluorescence shown in Figure 1A that ranges from cell membrane to parasites may suggest a relation to CD36, as CD36 reportedly internalizes holo-particles of HDL (Brundert et al., 2011), whereas SR-B1 selectively takes up the cholesteryl ester of HDL (Krieger, 1999).

HDL uptake by *Pb*-pRBCs was inhibited by SR-B1 antagonist BLT-1 (Nieland et al., 2002) (Figure 1D) in a dose-dependent manner (Figure 1E). BLT-1 also inhibited HDL uptake in CD36^+^, but SR-B1-deficient Huh7 cells (Supplementary Figure 2), showing that HDL uptake was mediated by SR-Bs. SR-B1-independent inhibition of HDL uptake was observed at 10 μM and this concentration clearly influenced both HDL uptake in *Pb*-pRBCs and *Pf* growth in *in vitro* culture supplemented with human serum (Figure 1E and Supplementary Figure 1C). The BLT-1 effects on parasitized erythrocyte may be due to off-target effects of BLT-1, but it also raises a possibility that other molecules may be influenced by the BLT-1 action in parasitized erythrocytes. To explore that possibility, we studied involvement of CD36 in *Pb* and *Pf* and compared with SR-BI.

### Identifications of Platelet-Derived Proteins on the Parasitized Erythrocytes

**FIGURE 2:**
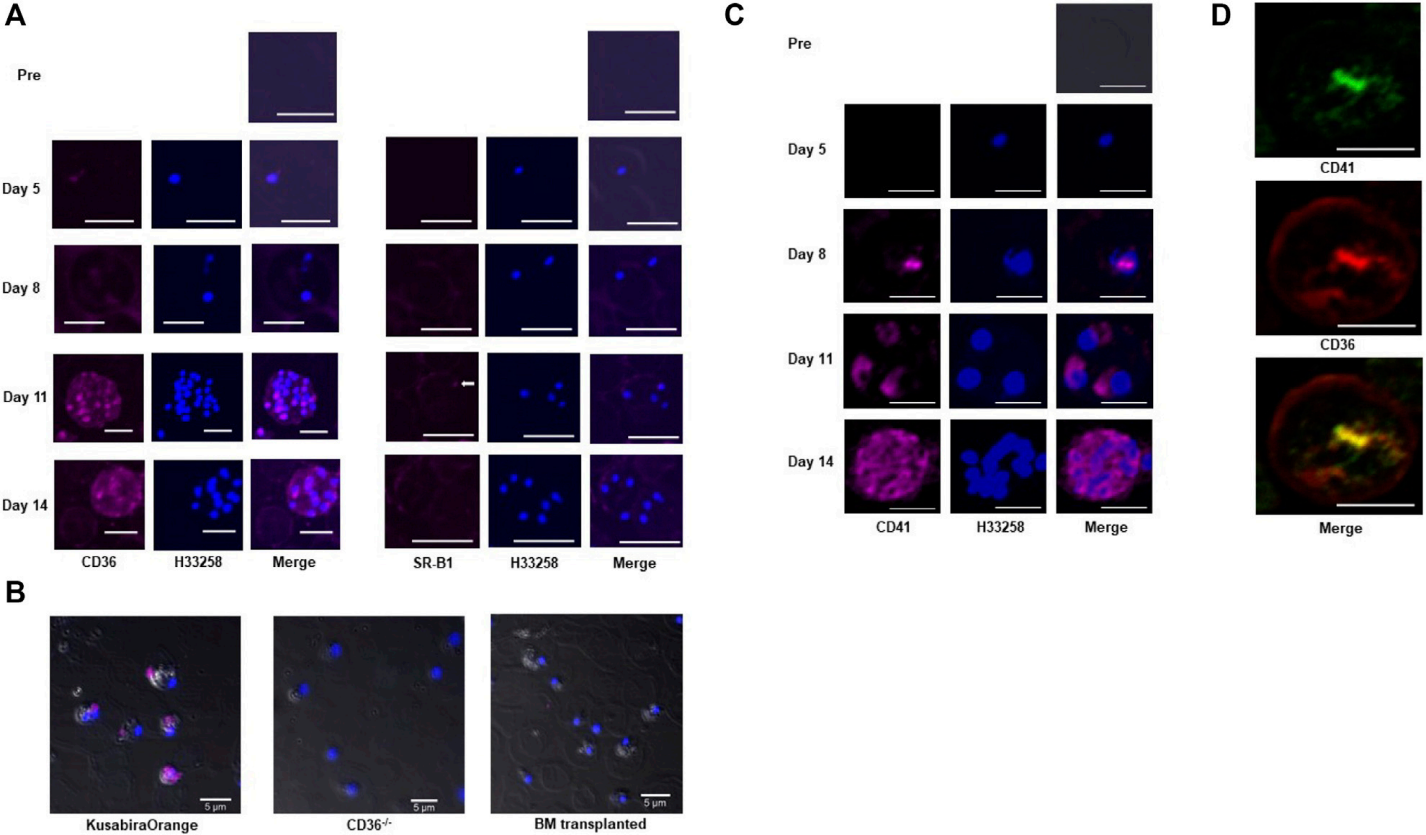
Immunocytochemical analysis of *Pb*-pRBCs for scavenger receptors and CD41. **(A)** Immunocytochemical analysis of *Pb*-pRBCs. The CD36 signals (pink) were weak in the pRBCs on days 5 and 8 (ring stage), but were abundantly detected on days 11 and 14 (schizonts). On day 11, CD36 appeared as a spot corresponding to each nucleus in the schizont’s body. SR-B1, which was detected in the pRBC membrane, was negligible in the internal parasites (arrow). Scale bar: 5 μm **(B)** Immunocytochemical analysis of CD36 in BM-transplanted mouse erythrocytes 6 days after *Pb* infection. CD36 (pink) was detected in pRBCs from the Kusabira-Orange mouse, but not in the BM-transplanted or CD36^−/−^ mouse. Scale bar: 5 μm. **(C)** Immunocytochemical analysis of pRBCs. CD41 (pink) signals were absent on days 5 and 8 (ring stage), but a small signal emanated from the internal parasites on day 8. CD41-specific fluorescence became abundant on days 11 and 14 (schizont stage). On day 11, CD41 appeared as spots corresponding to nuclei in the schizont’s body, as was also seen with CD36. Scale bar: 5 μm. **(D)** Co-localization of CD36 (red) and CD41 (green). Scale bar: 5 μm.

We investigated receptor protein levels on pRBCs over time in *Pb*-infected BALB/c mice because they usually survive malaria infections for more than 2 weeks. We collected blood from post-infection days 5–14. Up to day 5, most parasites we observed were ring forms, and very low levels of immunoreactive CD36 were observed. By day 11, most *Pb* parasites were schizonts, and CD36 signals appeared on the pRBC membranes and on parasites bodies (Figure 2A left). CD36 co-localized with apical membrane antigen 1 (AMA-1) (Supplementary Figure 3B), a *Plasmodium* integral membrane protein, suggesting that CD36 may be involved in sterol uptake from erythrocytes to the internalized parasites. Albeit the characteristic CD36 signal increased, the SR-B1 signal remained weak on the pRBCs, even on days 11–14 (Figure 2A right). CD36 was also detected in *Pf* culture supported by human serum (Supplementary Figure 3C).

The origin of this immunoreactive CD36 and how it is translocated to the pRBCs is unknown. To address this question, we performed bone marrow transplantations from CD36^−/−^ donor mice to congenic Kusabira-Orange (KuO) (Hamanaka et al., 2013) recipient mice. Fluorescent KuO is highly expressed in solid tissues, hematopoietic stem cells and five blood lineages (ending in neutrophils/macrophages, T-cells, B-cells, platelets, and erythrocytes) in the KuO mice, which enabled us to identify donor cells and distinguish them from recipient ones (Supplementary Figure 3D). Unlike the original KuO mice, the transplanted mice lost KuO fluorescence in their peripheral blood cells, including platelets, indicating that most recipient KuO blood cells were replaced by donor CD36^−/−^ blood cells. After *Pb* infection, CD36 was only detected on pRBCs from the KuO mice but not CD36^−/−^ mice or the transplanted mice (Figure 2B). This suggests that “immunoreactive CD36” was not derived from *Pb*, but came from the hematopoietic cells of the host mice. That CD36 is scarcely distributed on nRBCs provides clues about the specific CD36 translocation pathway for targeting parasitized cells. Among the hematopoietic cells, platelets and leukocytes (particularly those from the monocyte series) are known CD36-positive cells; thus, CD36 should originate from these cells. These observations motivated us to study CD41, a platelet and megakaryocyte marker, on pRBCs to discover the source of CD36.

Immunocytochemical analysis of the pRBCs from BALB/c mice revealed that the CD41 distribution was very similar to that of CD36, such that they often co-localized but with slightly different accumulation patterns (Figures 2C,D). CD41 signals also originated from the parasites within *Pf*-infected erythrocytes. CD41 on each parasite was especially evident in schizonts (Supplementary Figure 3E). These data support our hypothesis that molecules from platelets can be transferred to pRBCs, but with an unknown mode of transfer.

### Delivery of CD36 to Parasitized Erythrocytes by Exosomes From Platelets

**FIGURE 3:**
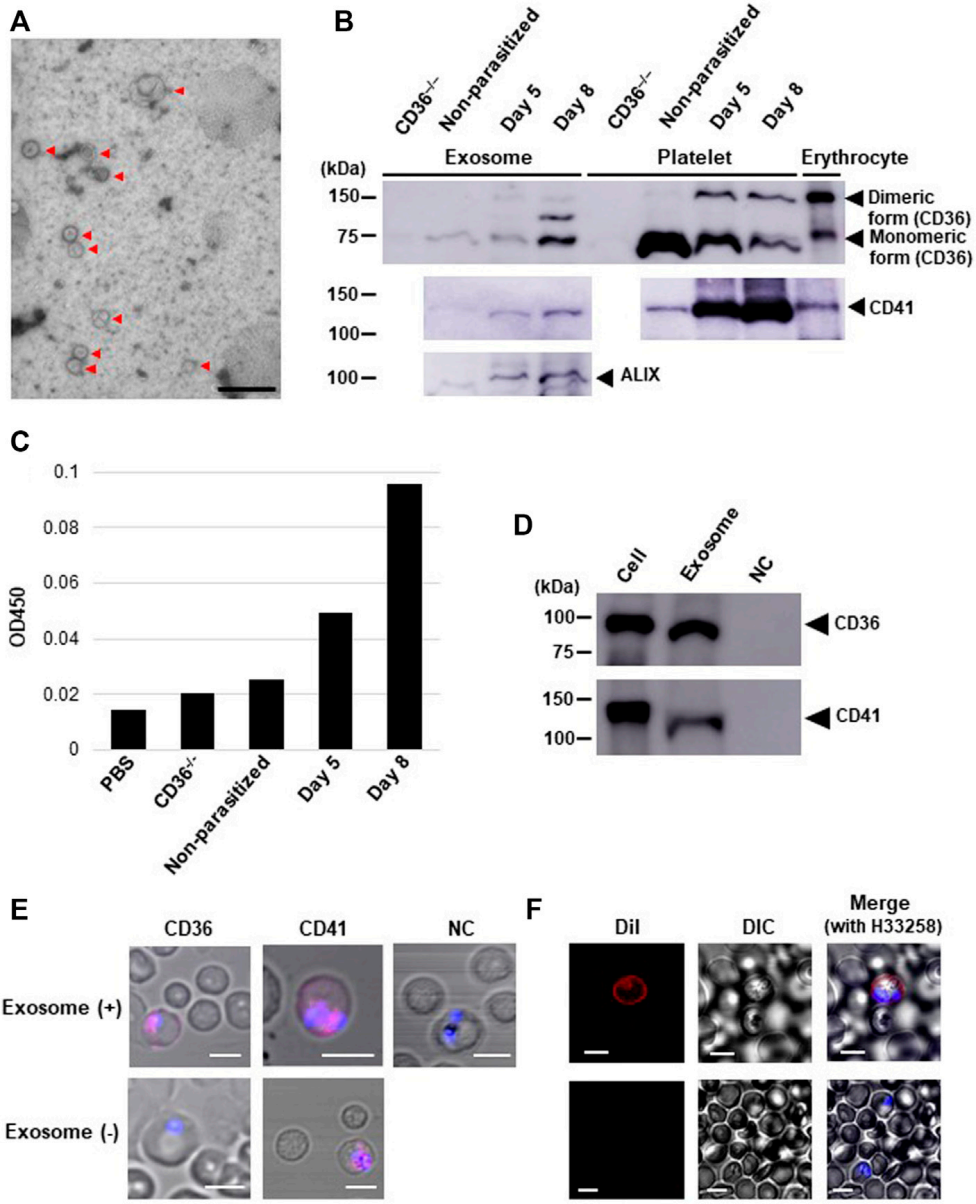
Analyses of the exosomes isolated from *Pb*-parasitized mouse plasma or from cultured cells differentiated from CMK11-5 cells. **(A)** Electron micrograph of the plasma exosomes (arrows) on day 8 post-infection. Scale bar: 333 nm. **(B)** Western blot of exosomes from plasma, platelets, and pRBCs. CD36, CD41 and an exosome marker (ALIX) were detected on days 5 and 8 post-infection. **(C)** Detection of exosomes doubly-positive for CD36 and CD41 by sandwich ELISA (duplicated). Each group of exosomes was isolated from the ‘pooled plasma’ of five mice. PBS and exosomes from a CD36^−/−^ mouse were used as negative controls. On days 5 and 8 post-infection, increased OD450 nm was observed. **(D)** immunoblot analysis for CD36 and CD41 in cultured cells differentiated from CMK11-5 cells or the exosomes released from those cells. **(E)** Immunocytochemical analysis of erythrocytes from a *Pb*-parasitized CD36^−/−^ mouse 6 h after adding CMK11-5-derived exosomes. CD36 and CD41 were detected from internal *Pb* (pink). NC: parasitized erythrocytes incubated with CMK11-5-derived exosomes and immunostained without primary Abs but with secondary Abs. Scale bar: 5 μm. **(F)** Trafficking of DiI-labelled exosomes derived from CMK11-5 cells into erythrocytes 1 h after exosome addition. DiI was observed in pRBCs (red) but not in uninfected ones. Scale bar: 5 μm.

Exosomes released from isolated human platelets carry CD41 (Aatonen et al., 2014). Our immunoblotting analysis showed increased CD36 and CD41 levels in the exosomes isolated from parasitized mouse plasma (Figure 3A), but not from non-parasitized mouse plasma, and they both had identical molecular sizes when isolated from platelets (Figure 3B). Parasite infection also resulted in appearance of dimeric forms and an additional band in exosome sample between monomeric and dimeric CD36, which may be a complex with other proteins occurred during the release of exosomes. To determine whether the CD36 molecules were platelet derived, we performed a sandwich enzyme-linked immunosorbent assay (ELISA) with exosomes from pooled mice plasma and observed increased levels of CD36–CD41 double-positive particles in the exosome fraction from post-infection days 5 and 8 (Figure 3C), suggesting that some CD36-positive exosomes were released from platelets in the parasitized mice. CD36 translocation from platelets to pRBCs was also observed by adding isolated exosomes collected from cultured CMK11-5 (a megakaryoblastic cell line which yields mature megakaryocytes by differentiation with phorbol 12-myristate 13-acetate, PMA) cells (Nagano et al., 1992) (Figure 3D) to the CD36^−/−^ mouse derived pRBCs (Figure 3E). Adding DiI-labelled exosomes to the parasite culture resulted in only pRBCs being labelled, with nRBCs remaining unlabeled (Figure 3F). These data show that platelet-derived CD36 was released as an exosome protein and captured by pRBCs but not by normal erythrocytes.

### CD36 May Be Involved in the Fitness of HDL Uptake *in vivo*

**FIGURE 4:**
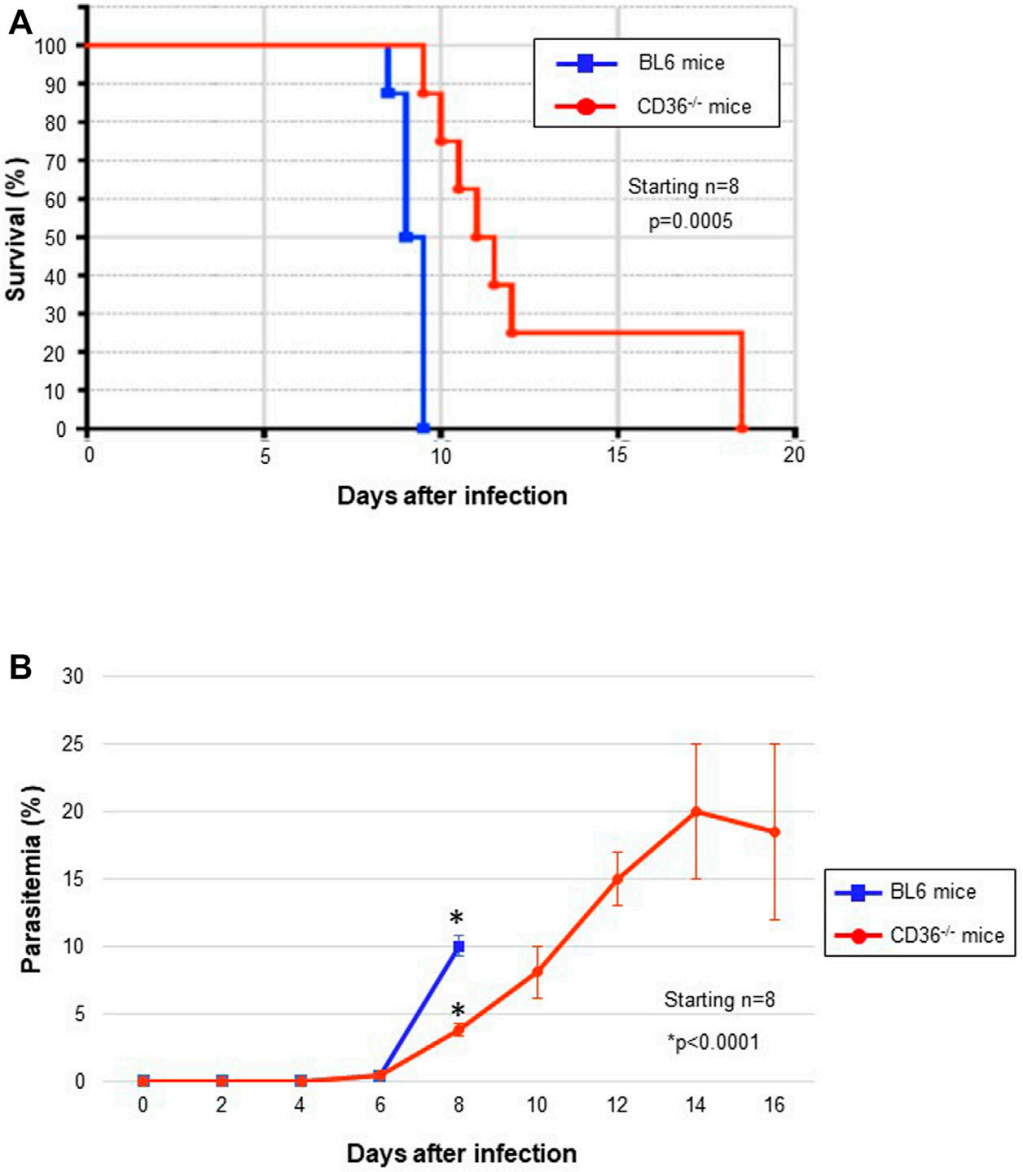
*In vivo Pb* survival and growth (parasitemia). **(A)** Kaplan-Meier survival curve of *Pb*-infected mice. CD36^−/−^ mice survived for significantly longer than the C57BL6 mice. **(B)** Parasitemia during *Pb* infection.

To study the involvement of CD36 in the pathogenesis of *Pb* infection, we compared parasitemia in normal C57BL6 and the CD36^−/−^ mice infected with ANKA strain. All infected BL6 mice died rapidly before day 10, but CD36^−/−^ survived longer than their congenic counterpart controls, although they eventually died at day 17. Parasitemia in the normal mice rapidly increased between days 6 and 8 post-infection, just before death, but parasitemia in the survived CD36^−/−^ mice continued to increase at slower rate (Figure 4A). These data may suggest that CD36-mediated HDL uptake is important for parasite growth and their pathogenesis, but other molecules partially compensates for HDL uptake in the CD36^−/−^.

## Discussion

Our data has shown that parasitized erythrocytes can take up cholesteryl esters and phospholipid from plasma HDL. The uptake of HDL by parasitized erythrocyte is mediated by CD36 and SR-BI that are acquired from platelet-derived exosomes through the interaction with erythrocytes. These results show an example of parasite survival skills to hijack host nutrients and proteins for efficient propagations.

Translocations of cholesterol from lipoproteins and extracellular space to erythrocytes could occur in the form of cholesteryl ester and free cholesterol (Ohkawa et al., 2020). Free cholesterol can enter erythrocytes when cholesterol-containing structures, such as HDL or simply in a vesicle form, physically contact erythrocyte membrane. A good example is a study by Plochberger et al., showing that ‘forced’ physical contact of cholesterol-preloaded HDL by atomic force microscope probe allowed only free cholesterol, but not cholesteryl ester, entered target membrane (Plochberger et al., 2017). This study suggests that hydrophobic nature of cholesterol allows spontaneous translocation between two membranes which might be due to the thermal fluctuations of cholesterol molecules. Our previous study by fluorescence lifetime microscopy study also showed that cholesterol-depleted erythrocytes by methyl-*β*-cyclodextrin treatment recovered cholesterol concentration by simply incubating them in human serum, indicating passive translocation of cholesterol to lipid membrane can slowly occur: it required more than 48 h to fully recovered to normal cholesterol level (Tokumasu et al., 2014). By contrast, receptor mediated incorporation can capture the whole lipoproteins, allowing both cholesteryl esters and free cholesterol can go into erythrocytes. However, significant reduction in parasite infection by cholesterol depletion from erythrocytes (Samuel et al., 2001) and the slow cholesterol recovery suggest that passive translocations of free cholesterol into erythrocytes may not be able to support normal parasite growth. We believe that aided translocation of HDL cholesterol should satisfy the needs of cholesterol by parasite.

The following three questions, however, remain unanswered: Q1: Why do platelet-derived exosomes increase in number in host plasma after *Plasmodium* infection? Q2: Why do platelet-derived exosomes prefer pRBCs over uninfected ones? Q3: How is CD36 trafficked to the internally-located parasites from or through the plasma membrane of pRBCs? To answer Q1, perhaps exosome release is explained by platelet activation although systemic platelet activation during parasite infection is still controversial as it may differ due to the parasite type and severity of disease (Kho et al., 2018). We did, in fact, collect the exosomes released from cultured CMK11-5 cells by adding CaCl_2_ and A23187 (a Ca^2+^ ionophore) to the culture media. Platelet activation and its resultant thrombocytopenia are inevitable in patients with malaria, and this favors parasite growth.

In answer to Q2, selective delivery to pRBCs possibly involves cytoadherence molecules. In *Pf*, *Pf*EMP1 mediates cytoadherence by binding to CD36 on endothelial cells (Gamain et al., 2001). *Pf*EMP1 may help with exosome capture and cause CD36 transfer to pRBCs. Cytoadherence through CD36 has also been reported in *Pb* (Franke-Fayard et al., 2005), although *Pf*EMP1 orthologues have not been identified in *Pb*. pRBCs may express other molecules that mediate adherence to platelet-derived exosomes. CD41, along with CD61, is known to form the α2b/β3 integrin complex (Blair and Frelinger, 2020), that is highly affinitive for many cytoadherence molecules. Our preliminary study showed that exosomes isolated from PMA-activated CMK11-5 cells were also taken up by mouse peritoneal macrophages, but CD41-negative exosomes from non-differentiated CMK11-5 were less taken up by peritoneal macrophages than CD41-positive exosomes. (Data not shown). This suggests that CD41 or α2b/β3 integrin complex expressed on the platelet-derived exosomes may contribute to cell binding. However, additional critical molecule(s) must be involved in the selective exosome delivery as CD41/CD61-deficient platelets still bind to parasitized erythrocytes (Pain et al., 2001). A possible candidate may be CD31, or PECAM-1 which can bind to *Pf*EMP-1 through DBLδ (Treutiger et al., 1997). Our data show that both differentiation-induced CMK11-5 and exosomes from these cells were positive for PECAM-1, and *Pb*-infected mouse erythrocytes were also PECAM-1 positive (Supplementary Figure 4), while non-parasitized erythrocyte were negative for PECAM-1. Incubation of *Pb*-infected mouse erythrocytes with CMK11-5*-*derived exosomes resulted in a higher PECAM-1 signal level, but non-parasitized erythrocytes remained negative even after incubation with the exosomes. PECAM-1 on exosomes may be recognized by parasite cytoadhering molecule(s) and that help capture exosomes.

A reasonable answer to Q3 may be that CD36 is translocated to intracellular parasites as internalized exosomes or exosome-derived microparticles from the plasma membrane of pRBCs. However, the DiI signal pattern and intensity on host erythrocytes varied. We believe that the heterogeneous nature of the erythrocyte population, which varies across erythrocyte ages, may influence the capture process involving both receptor-ligand interactions and the physical properties of the cell membranes, such as membrane fusion. Two hypotheses about how macromolecules are trafficked to protozoans within mammalian cells have been proposed, such as by forming channel-like membrane networks, or by host cell endocytosis (Robibaro et al., 2001). Our data may support both hypotheses. We observed two DiI signal patterns; positive for both protozoans and pRBC, or positive for only protozoans. The former may be resultant from exosome fusion with erythrocyte membranes, and the latter, direct endocytotic internalization of exosomes.

CD36 translocation reportedly occurs between the intracellular pool and the plasma membrane in skeletal muscle cells, a process stimulated by insulin and mediated by PI3K signaling (van Oort et al., 2008). Further investigation using PI3K inhibitors may help to elucidate the mechanism underlying CD36 trafficking.

In the course of searching responsible protein on the surface of parasitized erythrocytes, we used BLT-1 to study if additional scavenger receptors might be involved in the cholesterol take up. Our experiment showed BLT-1 inhibit HDL take up in SR-B1 deficient cells. We don’t exclude a possibility that this effect and inhibition of parasite growth we observed are partly due to the off-target effect of BLT-1 and toxicity from the copper-chelating activity of BLT-1 (Raldua and Babin, 2007). This should be considered in future studies using BLT-1 or other inhibitors. Another question for the BLT-1 effect is whether BLT-1 directly inhibit receptors on the parasitized erythrocyte for HDL take up, or it inhibits exosome binding to the parasitized erythrocytes. It may be technically difficult to answer this question because an experiment using pre-mixed BLT-1 and exosome before applying to parasitized erythrocytes would confuse data from the concurrent effect of ‘free’ BLT-1 that might occur on the erythrocyte surface receptors. However, the number of available receptors should be reduced by BLT-1 anyway, since the receptors being trafficked to the parasitized erythrocytes already exist on exosomes. More details of specific interaction of BLT-1with other proteins involved in parasitized erythrocyte metabolism remain to be studied.

**FIGURE 5:**
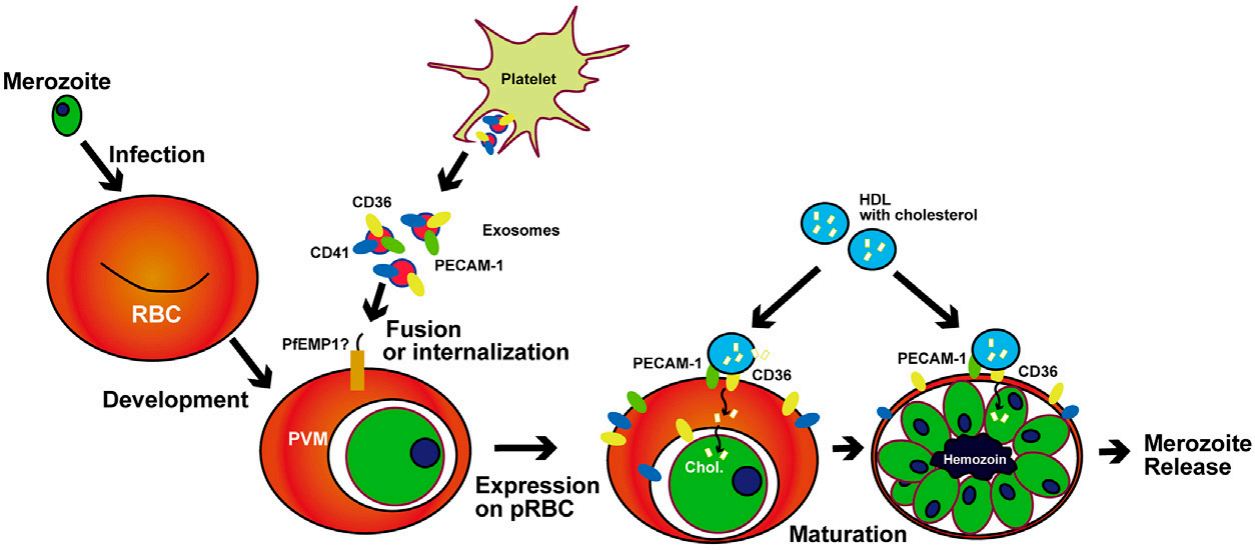
Proposed mechanism of exosome-based CD36 delivery and HDL capture in pRBCs. *Pf*EMP1 in *P. falciparum* and proteins that have similar binding ability to CD36 in mice malaria catch exosomes to hijack the receptors.

In summary, our findings revealed a notable and important aspect of host–parasite communication whereby malaria parasites hijack the exosome delivery of host scavenger receptors and lipids to support their own growth (Figure 5). Further study of this communication system may open the door to novel antimalarial therapies.

## Data Availability

The original contributions presented in the study are included in the article/Supplementary Material, further inquiries can be directed to the corresponding author/s.
