# Supplementary material for: Malaria Parasites Hijack Host Receptors From Exosomes to Capture Lipoproteins
Source: Front Cell Dev Biol. 2021 Nov 11;9:749153. doi: 10.3389/fcell.2021.749153 (PMC8631964; doi:10.3389/fcell.2021.749153)
Supplement: Supplementary file 1 [file DataSheet1.docx]

Supplementary Material

## Supplementary Figure 1


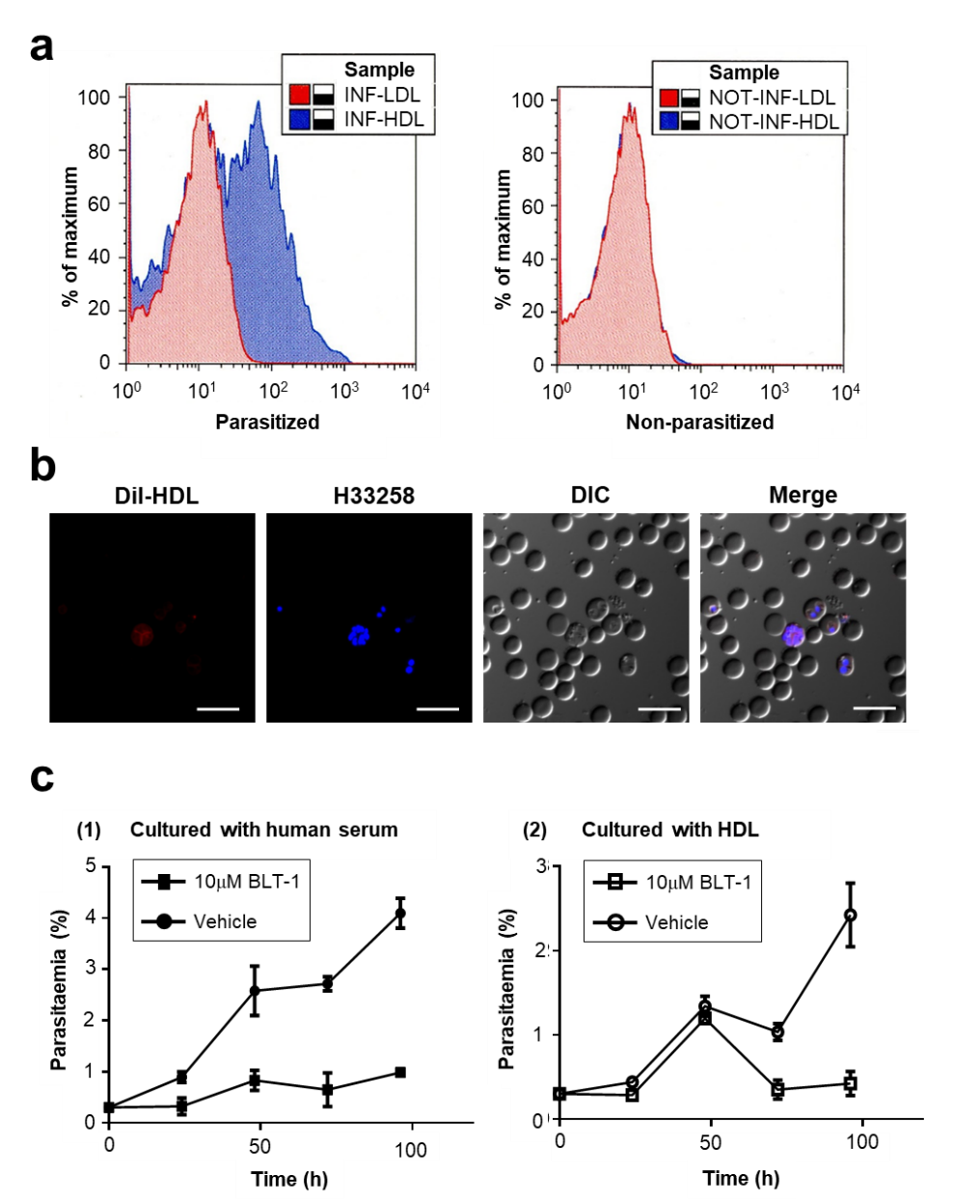


- **Supplementary Figure 1.** **a**, Flow cytometric analysis of DiO-labelled lipoproteins (red: LDL, blue: HDL) taken up by parasitized or nRBCs. HDL was taken up by pRBCs, but LDL was not. **b**, Analysis of DiI-labelled HDL uptake by *Plasmodium* *falciparum*-pRBCs. After 24h-incubation, DiI signalling was observed in the parasites. Scale bar: 10 μm. **c**, Growth inhibition of *Pf* by BLT-1 *in vitro*. Synchronous parasite culture was incubated 24, 48, 72, 96 h with DMSO or 10 µM BLT-1 under different serum conditions and the parasitaemia measured by Giemsa staining. (1) Closed circles and squares indicate parasites cultured in 10% human serum with DMSO or 10 µM BLT-1, respectively. (2) Open circles and squares indicate parasite cultures containing 500 µg/mL HDL with DMSO or 10 µM BLT-1, respectively.

**Supplementary Figure 2**

**
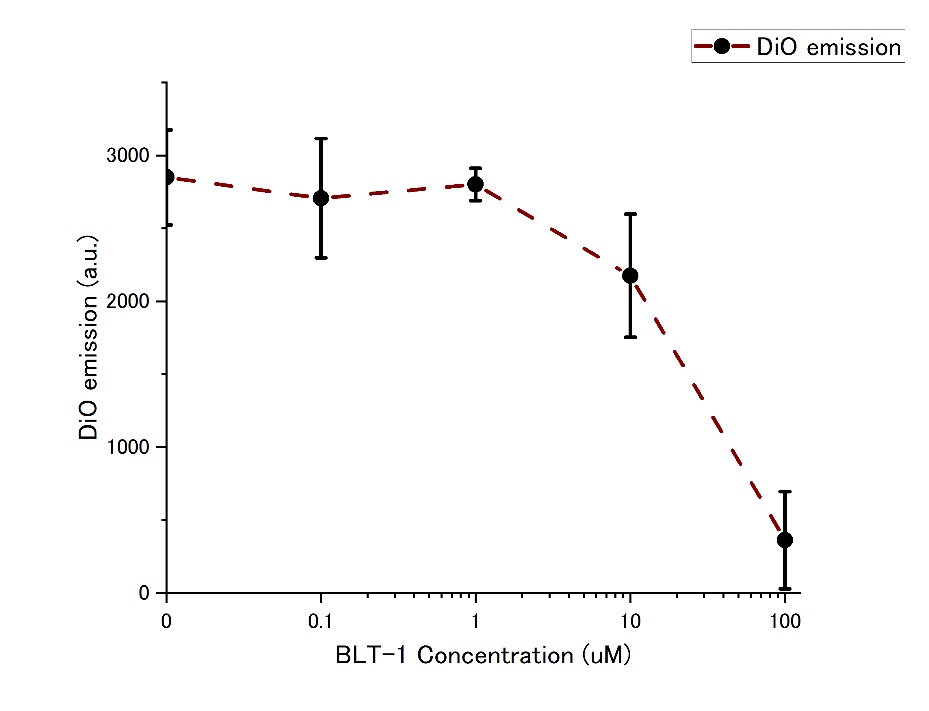
**

**Supplementary Figure 2.** HDL uptake in SR-B1-deficient Huh-7 cells (CD36-positive cells) was inhibited by BLT-1 in a dose-dependent manner. SR-B1-deficient HuH-7 cells were preincubated for 1 h with different concentrations of BLT-1, followed by incubation for 8 h with DiO-labelled HDL. The DiO signal was measured at 485 nm (ex.)/520 nm (em.) to quantify HDL uptake. Error bar: SEM (n=6).

**
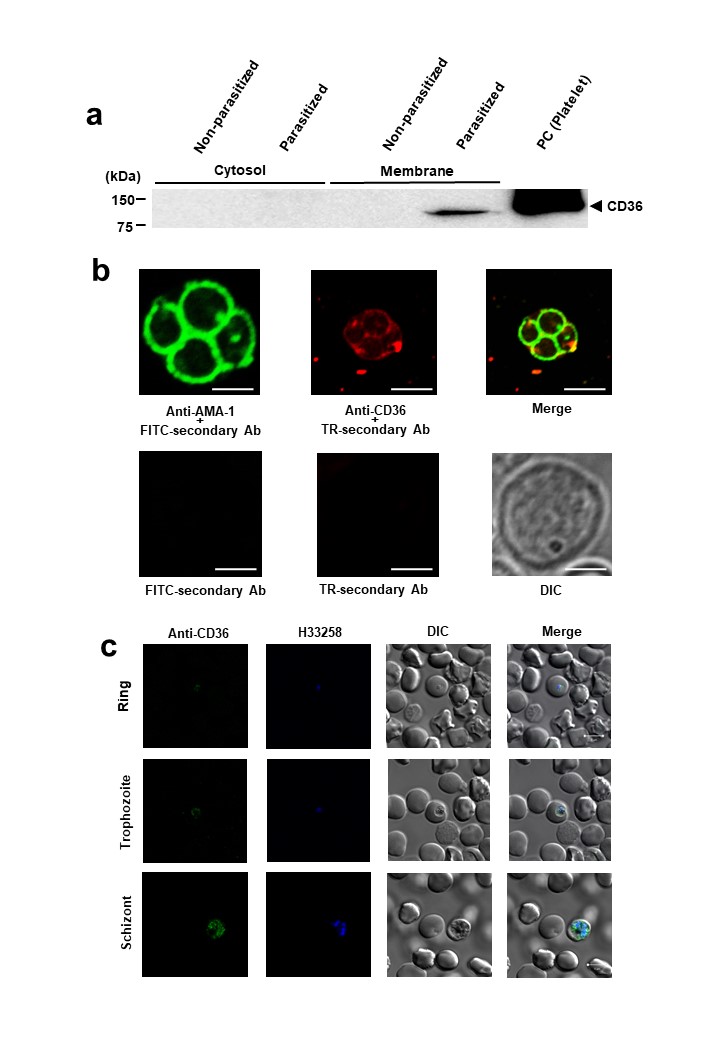
Supplementary Figure 3**

**
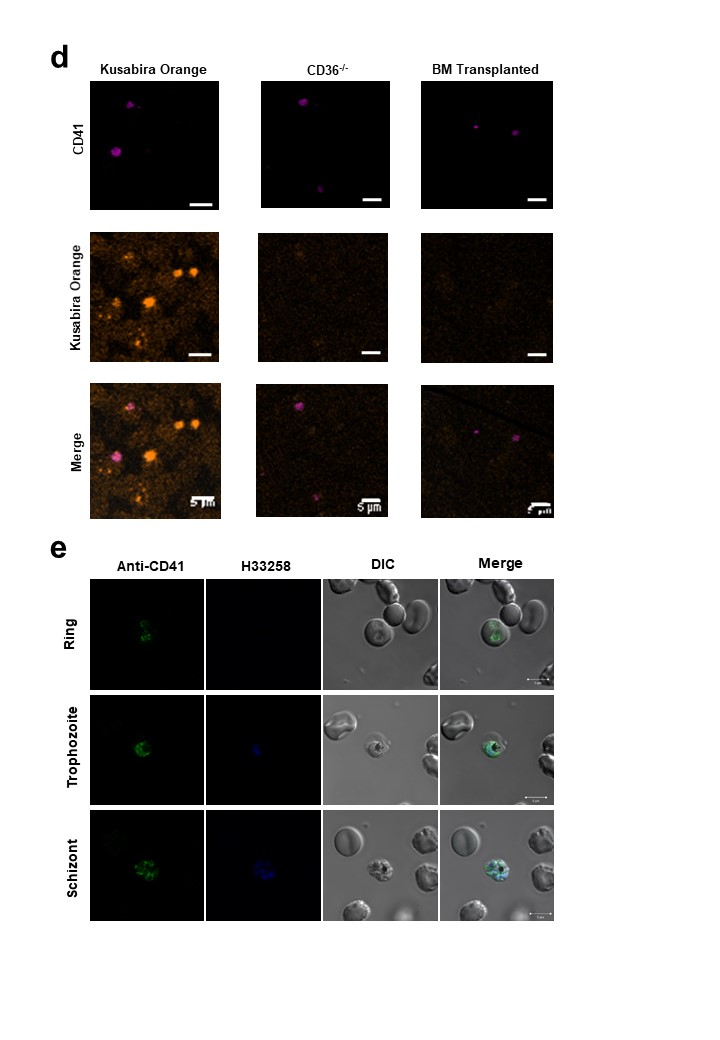
**

**Supplementary Figure 3. a**, Western blot of *Pf*-pRBCs. CD36 was detected in the membrane fraction of these erythrocytes. **b**, Co-localization of CD36 (red) and AMA-1 (green). AMA-1 is a marker for the apical membrane of *Pb* parasites. The lower photos show the negative controls. Scale bar: 5 μm. **c**, Immunocytochemical analysis of *Pf*-infected erythrocytes for CD36 (green). Scale bar: 5 μm. **d**, Kusabira-Orange (KuO) mouse with bone marrow transplanted from a CD36^–/–^ mouse prior to *Pb* infection. No Kusabira-Orange fluorescence (orange) signal was detected in platelets from the transplanted mouse (pink), suggesting that the recipient-derived platelets were replaced by donor-derived ones. Scale bar: 5 μm. **e**, Immunocytochemical analysis of *Pf*-infected erythrocytes for CD41 (green). Scale bar: 5 μm.

**Supplementary Figure 4**


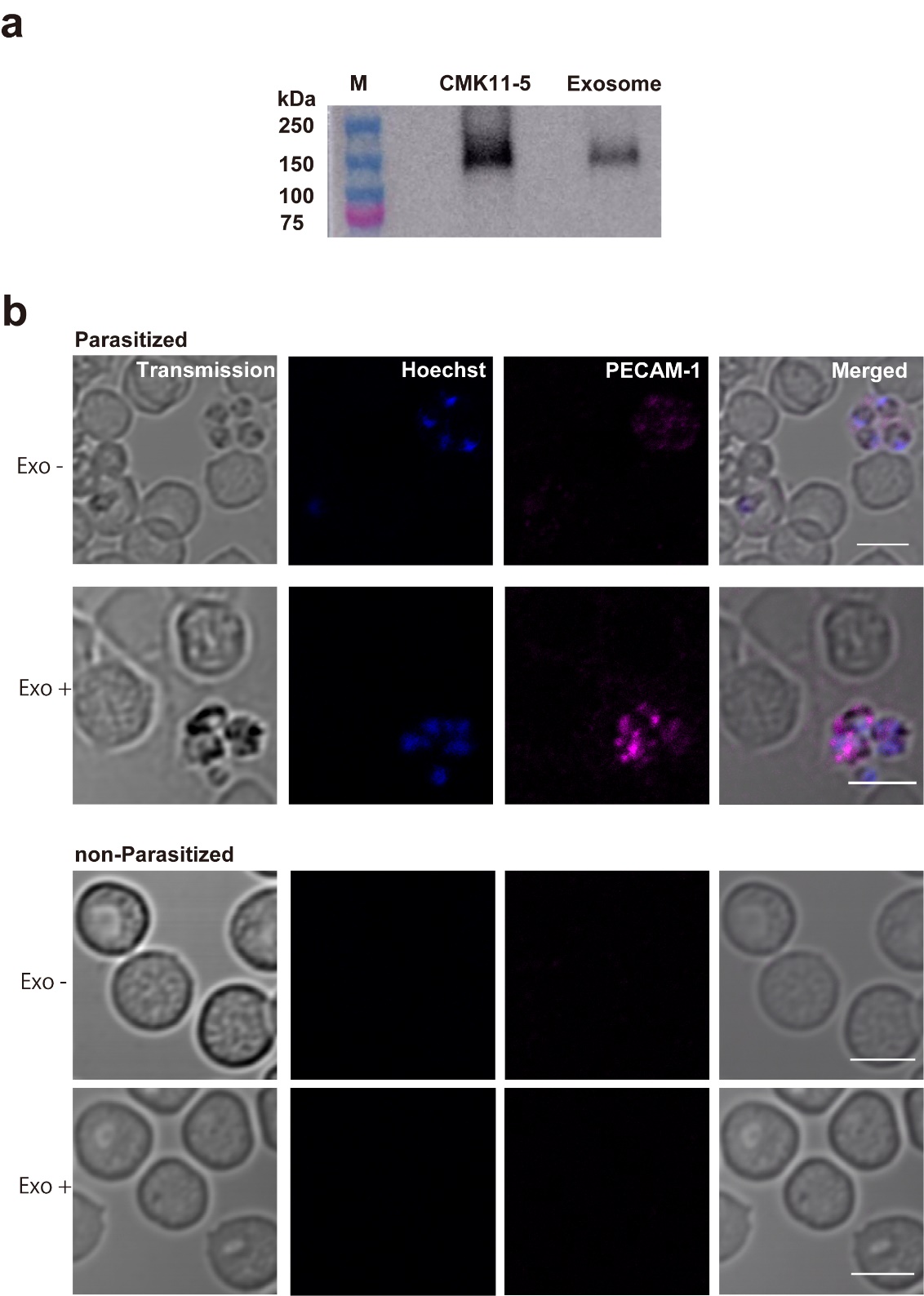


**Supplementary Figure 4. a**, Immunoblot analysis for PECAM-1 in cultured cells differentiated from CMK11-5 cells or the exosomes released from those cells. 5μg as protein was applied for each lane.

**b**, Immunocytochemical analysis of erythrocytes from a *Pb*-parasitized or a non-parasitized C57BL6 mouse 6h after adding CMK11-5-derived exosomes or not. PECAM-1 was detected from internal *Pb* (pink). The signal appeared more intense after adding exosomes. Scale bar: 5 μm.
